# Parasites and Infectious Diseases: Discovery by Serendipity and Otherwise

**DOI:** 10.3201/eid1403.071540

**Published:** 2008-03

**Authors:** C. Ben Beard

**Affiliations:** * Centers for Disease Control and Prevention, Fort Collins, Colorado, USA

The author of this book retells several of the famous stories of discovery in the field of vector-borne and parasitic diseases through the eyes of some of the most prominent researchers working in this field today. But Esch does not stop there—he goes on to connect these early stories with more recent watershed contributions as recounted through a series of interviews he references throughout the course of the book. For example, he describes the discovery of the African sleeping sickness agent, *Trypanosoma gambiense*, and the contributions in the early 1900s by Dutton, Castellani, and Bruce. He then moves on to more recent discoveries regarding immunity, antigenic variation, and the role of variable surface glycoproteins. He describes the seminal studies that were performed in this area as recounted through interviews with prominent parasitologists Dick Seed and Keith Vickerman. Through this process, Esch weaves a tapestry of the new and old as it relates to the history of important tropical diseases such as African typanosomiasis, malaria, yellow fever, HIV/AIDS, hookworm, and schistosomiasis, which continue to plague humankind.

The book has a novel organization; the first 100 pages are devoted to a lengthy prologue in which the major content contributors, the disease experts Esch interviewed, are extensively quoted and even venerated, in a casual, entertaining, and well-deserving manner. Many who peruse this book will be pleasantly surprised by the colorful biographies of well-known parasitologists and vector-borne disease specialists, who some readers no doubt will know as friends, colleagues, collaborators, professors, or even former students.

As the title suggests, there is an effort to locate threads of serendipity woven through the historical tapestry of discovery. The conclusions that emerge unsurprisingly are that serendipity has played a much less important role than one might naively surmise and that the qualities of hard work, rock-solid persistence, a keen mind, and the fortitude to swim against the academic tide have paid generous dividends.

No doubt the attribution of specific recent hallmark contributions by key persons, many of whom are still active in the field today, will be met with some degree of controversy. Nevertheless, the book is interesting, educational, and enjoyable, a “must have” for every library of tropical medicine or medical history. Thumbs up to Esch.

